# Clinical performance of contrast-enhanced spectral mammography in pre-surgical evaluation of breast malignant lesions in dense breasts: a single center study

**DOI:** 10.1007/s10549-020-05881-2

**Published:** 2020-08-28

**Authors:** Anna Bozzini, Luca Nicosia, Giancarlo Pruneri, Patrick Maisonneuve, Lorenza Meneghetti, Giuseppe Renne, Andrea Vingiani, Enrico Cassano, Mauro Giuseppe Mastropasqua

**Affiliations:** 1 Division of Breast Radiology, IEO, European Institute of Oncology IRCCS, Via G.Ripamonti, 435, 20141 Milan, Italy; 2 School of Medicine, University of Milan, Milan, Italy; 3 Department of Pathology, Fondazione IRCCS Istituto Nazionali Tumori Milano, Via G. Venezian, 1, 20133 Milan, Italy; 4 Division of Epidemiology and Biostatistics, IEO, European Institute of Oncology IRCCS, Via G. Ripamonti, 435, 20141 Milan, Italy; 5 Division of Pathology and Laboratory Medicine, IEO, European Institute of Oncology IRCCS, Via G. Ripamonti, 435, 20141 Milan, Italy; 6 School of Medicine, University of Bari, Bari, Italy; 7 Department of Emergency and Organ Transplantations, Section of Anatomic Pathology, Piazza G. Cesare 11, 70124 Bari, Italy

**Keywords:** Contrast-enhanced spectral mammography, Ultrasound, Full field digital mammography, Magnetic resonance, Breast malignant lesions

## Abstract

**Purpose:**

To compare the efficacy of contrast-enhanced spectral mammography, with ultrasound, full field digital mammography and magnetic resonance imaging in detection and size estimation of histologically proven breast tumors.

**Methods:**

This open-label, single center, prospective study, included 160 dense breast women with at least one suspicious mammary lesion evaluated by ultrasound, full field digital mammography and magnetic resonance imaging in whom a mammary tumor was histologically proven after surgery performed at the European Institute of Oncology between January 2013 and December 2015. Following the complete diagnostic procedure, the patients were further investigated by contrast-enhanced spectral mammography prior to surgery.

**Results:**

Overall, the detection rate of malignant breast lesions (in situ and invasive) was 93.8% (165/176) for contrast-enhanced spectral mammography, 94.4% (168/178) for ultrasound, 85.5 (147/172) for full field digital mammography and 97.7% (173/177) for magnetic resonance imaging. Radiological measurements were concordant with the post-surgical pathological measurements of the invasive tumor (i.e., within 5 mm) in: 64.6% for contrast-enhanced spectral mammography, 62.0% for ultrasound, 45.2% for full field digital mammography (*p* < 0.0001) and 69.9% for magnetic resonance imaging (*p* = 0.28); underestimated in: 17.4% for contrast-enhanced spectral mammography, 19.6% for ultrasound, 24.2% for full field digital mammography (*p* = 0.03) and 6.7% for magnetic resonance imaging (*p* = 0.0005); and overestimated in: 16.2% for contrast-enhanced spectral mammography, 16.6% for ultrasound, 16.6% for full field digital mammography and 22.7% for magnetic resonance imaging (*p* = 0.02).

**Conclusions:**

Our data suggest that contrast-enhanced spectral mammography improves on full field digital mammography and is comparable to ultrasound and magnetic resonance imaging in terms of detection sensitivity and size estimation of malignant lesions in dense breasts.

## Introduction

Contrast-enhanced spectral mammography (CESM) is a recently developed imaging technique relying on visualization of iodinated contrast agent uptake, proposed as a new complementary approach to breast imaging [1, 2]. At present, breast cancer (BC) diagnosis and staging rely mainly on three diagnostic techniques: full field digital mammography (FFDM), ultrasound (US), mammography and magnetic resonance imaging (MRI). FFDM is best suited to studying breast tissue with a predominantly adipose component [1, 2] as its lower sensitivity in dense, fibro-glandular breasts may lead to increased false negative rates and cancer size underestimation, especially in young patients [3, 4]. US, on the other hand, is particularly suitable for studying dense breasts, but its overall diagnostic efficacy is affected by the high false positive rate [3]. MRI is characterized by high sensitivity, independent of breast density, but its specificity is limited by high (up to 19%) false positive rates [5]. Moreover, evidence suggests that MRI is associated with treatment delay without an associated improvement in margin clearance at surgery and clinical outcome [6].

In this scenario, CESM, which is similar to MRI in taking advantage of the differential enhancement between neoplastic and normal tissue, has recently gained momentum as an innovative and clinically useful method for preoperative breast assessment [7–10].

In CESM, a pair of images are acquired for each view: one low-energy image (LE), similar to a standard mammogram, and one high-energy image (HE), which is optimized for the detection of iodine contrast agent uptake; the two images are then combined to produce an image where glandular tissue texture is suppressed and contrast uptake is highlighted. Exposure techniques for acquisition of LE CESM images are similar to those used for conventional digital mammography.

In particular, CESM has better sensitivity without a loss of specificity compared with mammography alone and improves lesion delineation through visualization of contrast medium uptake within breast lesions. There is an increased cancer detection rate for CESM over MG, comparable to MRI, and also an improvement in size estimation [11–13].

In patients with dense breasts, it has been recently shown that CESM is more sensitive than traditional imaging techniques, irrespective of patient menopausal status, and shows significantly lower false positive rates than MRI and in some preliminary study of the last years CESM, can replace the standard mammogram in symptomatic women [7–15].

The main aim of our study was to compare CESM with conventional imaging techniques in pre-surgical breast assessment of women with dense breasts, using tumor size estimation as a pre-specified endpoint**.**

## Methods

### Patients

Ethics Committee approval was obtained for this retrospective study as well as patients’ informed consent.

The study population consisted of 160 women diagnosed and treated at the European Institute of Oncology in Milan, between January 2013 and December 2015. These patients were characterized as having dense breasts (ACR 3 and 4 on FFDM), and presented at least one suspicious mammary lesion evaluated by US, FFDM and MRI, subsequently confirmed as a malignant lesion (in situ or invasive) at histo-pathological evaluation of the surgical specimen. Prior to surgery, all the patients received a diagnosis of C4 or C5 by fine-needle aspiration, or a B4 or B5 diagnosis by core biopsy or vacuum-assisted (VABB) breast biopsy [16]. For a full listing of the inclusion and exclusion criteria, see Table 1.

**Table 1:** Inclusion criteria: Full list of the inclusion and exclusion criteria of the study

Eligibility criteria	
1. Previous digital mammography and ultrasound and magnetic resonance imaging	
2. Suspicious or malignant diagnosis by FNAC (C4 or C5), by core biopsy (CB) or VABB (B4 or B5)	
3. Dense breast (ACR class 3 or 4 on mammography)	
4. Capable and willing to comply with study procedures, having signed the informed consent form	
5. Breast size compatible with the dimensions of the image detector	
6. In sufficiently good health to undergo standard breast cancer care and CESM examination	
Exclusion criteria	
1. High risk of adverse events related to iodinated contrast agents	
2. Previously included in this study	
3. Participating in, or having participated in another clinical trial(s) within the 30 days precedent	
4. Previous breast reconstructive surgery or breast implant(s)	
5. Concomitant or previous radiotherapy to the thorax area or systemic chemotherapy	
6. Proven or supposed pregnancy	

All of the patients were examined pre-operatively using a CE-Marked, Senographe Essential, full field digital system, with SenoBright option (GE Healthcare, Chalfont St. Giles, UK), for dual-energy CESM acquisitions. In brief, this involved a catheter being placed into the antecubital vein of the arm contra-lateral to the suspicious breast lesion, and a one-shot intravenous injection of 1.5 ml/Kg of contrast agent (Visipaque® 320, GE) using a power injector. Two minutes after the contrast agent administration, bilateral cranio-caudal (CC) and mediolateral oblique (MLO) views were obtained with standard compression at 2 different energy exposures (high and low). For the high energy acquisitions, Molybdenum/Cupper or Rhodium/Cupper filters were used instead of traditional Molybdenum/Molybdenum, Rhodium/Rhodium or Molybdenum/Rhodium, which were used for low energy acquisitions. The low- and high-energy images were automatically combined by the scanner software to produce subtracted images reflecting contrast agent content. The total X-ray dose delivered to the patient was 20%-30% higher than that delivered for a standard digital mammogram, according to the thickness of the breast during the compression (4–10 N) [7]. The total examination time was roughly six minutes.

One breast radiologist, with 20 years of experience, blinded to the original reports of US, FFDM and MRI, evaluated the images of CESM.

Patients were surgically operated within 6 weeks of diagnosis. In keeping with the study goals of comparing CESM breast lesion detection rates with US, FFDM, and MRI, and of ascertaining their relative ability to estimate breast tumor size, the surgical pathology report was adopted as the reference standard. To this end, the surgical specimens were sampled and evaluated immediately after surgical excision, prior to formalin fixation, to avoid shrinkage due to the fixation process, and ultimately allowing a more reliable comparison with in vivo data. In cases of palpable, grossly visible DCIS lesions, the extent was reported as the greatest dimension at gross evaluation; in case of non-palpable lesions, the serial sequential sampling method was used, by mapping tissue blocks, as previously reported [17].

As regards lesion size, the results were coded as follows: negative (tumor not detected by test method); concordant (tumor detected and size was within 5 mm of pathology dimension); underestimated (tumor detected but size underestimated by < 5 mm); overestimated (tumor detected but size overestimated by > 5 mm).

We also performed a secondary analysis of possible interactions between detection rates and size estimation for each technique and traditional pathological characteristics (multifocality, histotypes, coexistence of an in situ component, histological grade, peritumoral vascular invasion, and pT), as possible confounding factors.

### Statistical methods

**Fig. 1:**
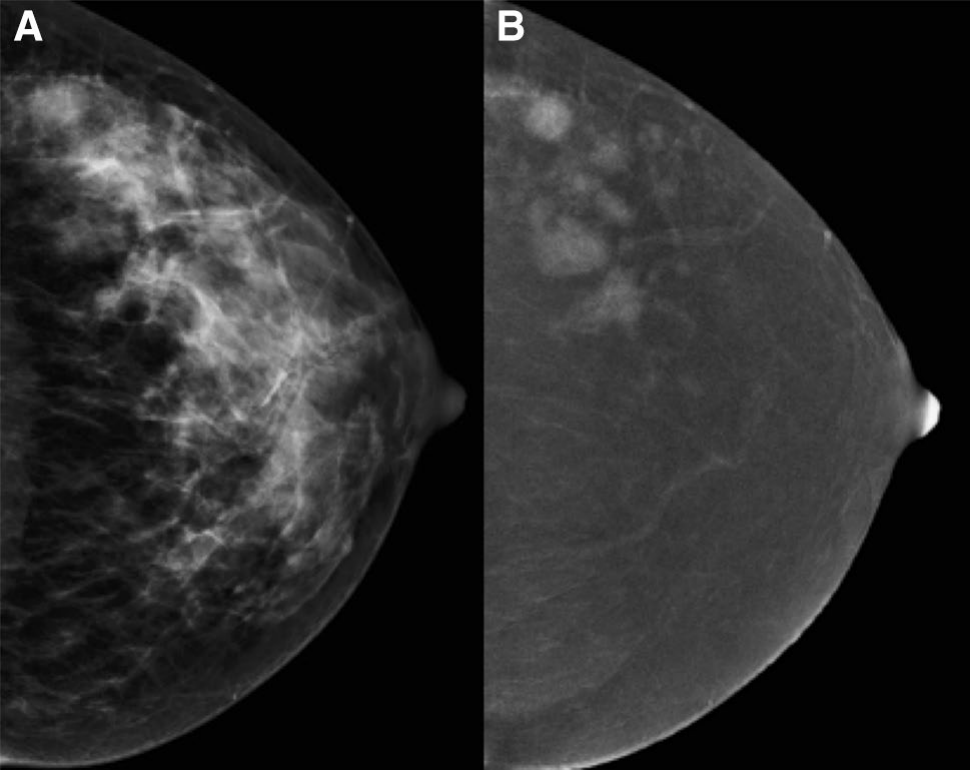
**a** CC FFDM projection showing a dense breast. **b** CC projection of the same breast on the CESM iodine content image revealing multiple neoplastic nodules

**Fig. 2:**
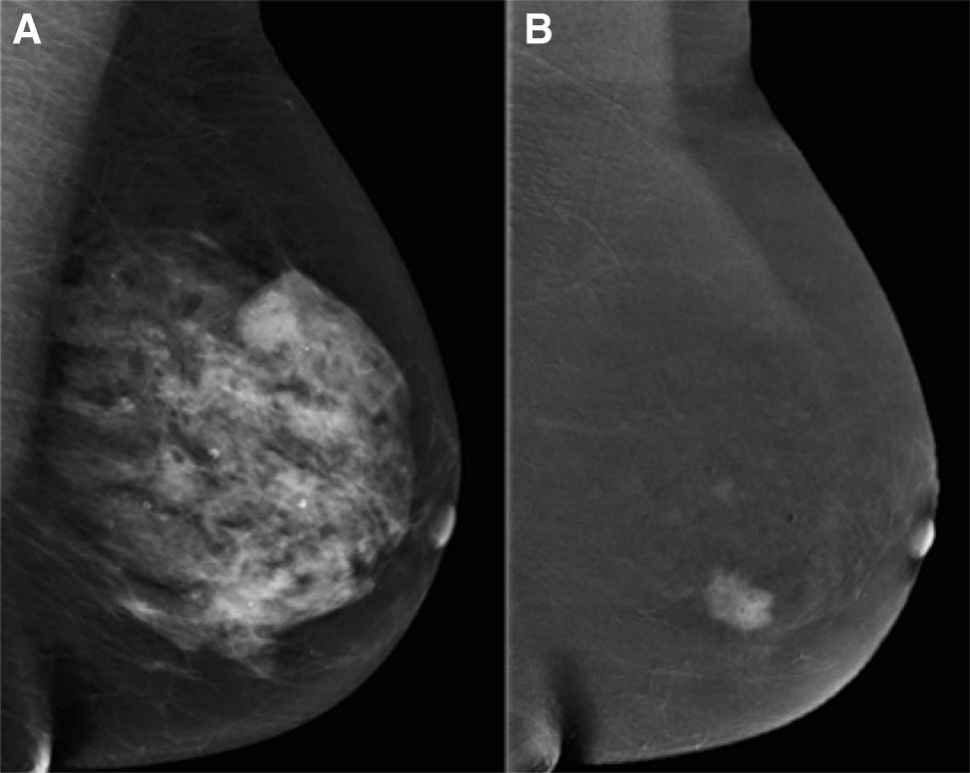
**a** MLO FFDM projection showing a dense breast. **b** MLO projection of the same breast on CESM, iodine content image revealing breast cancer

**Fig. 3:**
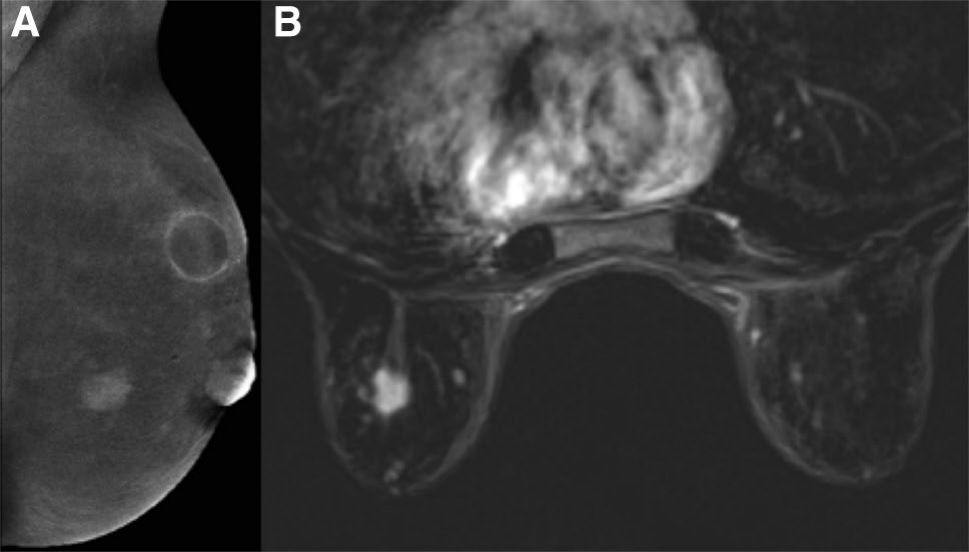
**a** MLO CESM iodine content image projection showing a principal centimeter diameter breast cancer within the inner quadrant, and a sub-centimetric satellite lesion. **b** The breast cancer and satellite nodule are seen in the contrast-enhanced MR image

**Fig. 4:**
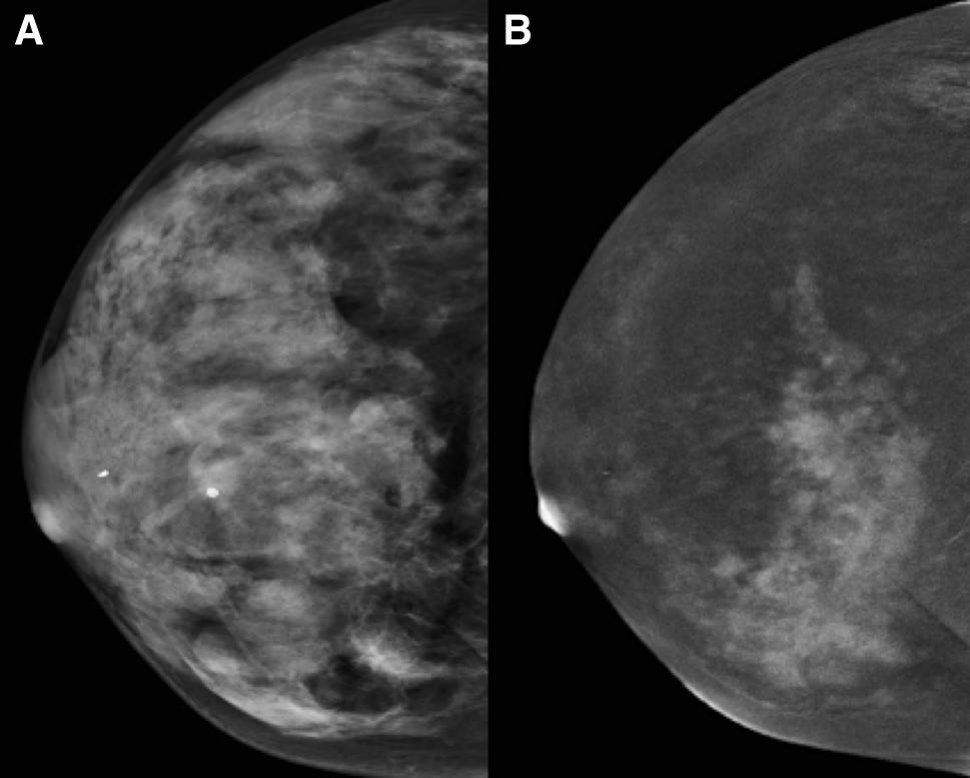
**a** CC FFDM projection showing a dense breast. **b** CC projection on CESM iodine content image showing diffuse breast cancer

The Clopper-Pearson exact method was used to estimate 95% confidence intervals for proportions. The McNemar test was used to assess the agreement of CESM findings with those of US, FFDM and MRI. The Fisher exact test was used to assess difference of results across subgroups of patients. Analyses were performed with SAS version 9.2 (Cary NC). All *p*-values are two-sided (Figs. 1, 2, 3, and 4).

## Results

Clinico-pathological characteristics of the 160 patients included in the study are detailed in Table 2. Post-surgery pathological examination revealed 178 breast lesions (163 invasive tumors and 15 non-invasive tumors). CESM, FFDM, and MRI were not performed in one and two and six cases of invasive tumor, respectively, due to peripheral location of the lesions or technical reasons.

**Table 2:** Patients characteristics

(a) Ages of patients included in the study	
	*N* (%)
Age (year)	
< 40	5 (3.1%)
40–49	71 (47.3%)
50–59	59 (36.9%)
60–69	16 (10%)
70 +	9 (5.6%)
< 50	76 (47.5%)
50 +	84 (52.5%)

(b) Patients characteristics^a^ (*N* = 160): Histology, receptor status, and staging	
	*N* (%)
pT (ypT)	
0	4 (2.5)
1mi	2 (1.2)
1a	24 (15.0)
1b	66 (41.2)
1c	2 (1.2)
2	46 (28.7)
3	14 (8.8)
4	1 (0.6)
Missing	1 (0.6)
pN	
0	83 (51.9)
1	42 (26.2)
2	12 (7.5)
3	16 (10.0)
X	6 (3.7)
Missing	1 (0.6)
Grade	
G1	27 (16.9)
G2	70 (43.7)
G3	57 (35.6)
Missing^b^	6 (3.7)
ER	
ER+	95
ER−	65
Missing^b^	0
PgR	
PgR+	95
PgR−	65
Missing^b^	0
Ki 67	
Ki 67 low (< 20%)	88
Ki 67 high (> 20%)	70
Missing^b^	2
Her2/neu	
* Her2/neu* +	90
* Her2/neu-*	67
Missing^b^	3

^a^Final characteristics of the worst nodule when more than one present per patient

^b^Or not evaluable

Overall, the detection rate of breast malignant lesions (invasive and in situ) was 93.8% for CESM, 94.4% for US, 85.5% for FFDM and 97.7% for MRI. In particular, the detection rate in invasive tumors was 98.1% for CESM, 98.2% for US (*p* = 1.00), 86.0% for FFDM (*p* < 0.0001) and 99.4% for MRI (*p* = 0.50) (Table 3). Somewhat lower were the detection rates for non-invasive tumors: 46.7% for CESM, 53.3% for US (*p* = 1.00), and 80.0% for both FFDM and MRI (*p* = 0.06) (Table 4).

**Table 3:** Detection rate and size estimation in invasive tumors

Lesions	US	FFDM	CESM	MRI
	163	157	161	163
(a) Detection rate versus pathology				
Lesions detected	160 98.2% (94.7–99.6%)	135 86.0% (79.6–91.0%)	158 98.1% (94.7–99.6%)	162 99.4% (96.6–100%)
Performance CESM	1.00	**< 0.0001**	–	0.50

	US	FFDM	CESM	MRI
(b) Lesion size versus pathology				
Negative	3 1.8% (0.4–5.3%)	22 14.0% (9.0–20.4%)	3 1.9% (0.4–5.4%)	1 0.6% (0.0–3.4%)
Performance CESM*	1.00	** < 0.0001**	-	0.50
Underestimated	32 19.6% (13.8–26.6%)	38 24.2% (17.7–31.7%)	28 17.4% (11.9–24.1%)	11 6.7 (3.4–11.8%)
Performance CESM*	0.56	**0.04**	–	**0.0005**
Concordant	101 62.0% (54.0–69.4)	71 45.2% (37.3–53.4%)	104 64.6% (56.7–72.0)	114 69.9% (62.3–78.9%)
Performance CESM*	0.56	** < 0.0001**	–	0.28
Overestimated	27 16.6% (11.2–23.2%)	26 16.6% (11.1–23.3%)	26 16.2% (10.8–22.8%)	37 22.7% (16.5–29.9%)
Performance CESM*	1.00	1.00	–	**0.02**

FFDM was not evaluable for 6 patients, CESM for 2 patients and MRI for 1 patient

*FFDM* full field digital mammography, *CESM* contrast enhanced spectral mammography, *US* ultrasound, *MRI* magnetic resonance imaging

The performance of CESM with respect to US, MRI and FFDM is evaluated with McNemar Test *p*-value

**Table 4:** Detection rate in intraductal tumors

	Lesions	Detection rate							
		US		FFDM		CESM		MRI	
		*N*	Rate (95% CI)	*N*	Rate (95% CI)	*N*	Rate (95% CI)	*N*	Rate (95% CI)
ALL	15	8	53% (27–79%)	12	80% (52–96%)	7	47% (21–73%)	12	80% (52–96%)
DIN1c/ (DCISG1)	4	2	50% (7–93%)	3	75% (19–79%)	1	25% (1–81%)	3	75% (19–79%)
DIN2/ (DCISG2)	7	5	71% (29–96%)	6	86% (42–100%)	3	43% (10–82%)	6	86% (42–100%)
DIN3/ (DCISG3)	4	1	25% (1–81%)	3	75% (19–79%)	3	75% (19–79%)	3	75% (19–79%)
Performance wrt CESM^a^		1.00		0.06		–		0.06	

^a^*wrt* with respect to

*FFDM* full field digital mammography, *CESM* contrast enhanced spectral mammography, *US* ultrasound, *MRI* magnetic resonance imaging, *LIN* lobular intraepithelial neoplasia, *DIN* ductal intraepithelial neoplasia

The performance of CESM with respect to US, MRI and FFDM is evaluated with McNemar Test *p*-value

Size estimation was evaluated only for the histologically proven invasive tumors (Table 3). The histological diagnoses were invasive ductal carcinoma NST (131), invasive lobular carcinoma (21), acinic carcinoma (2), mixed ductal–lobular carcinoma (8) and malignant phyllodes tumor (1). The radiological final pathological measurement of size in the 163 invasive lesions were concordant in 64.6% (104) for CESM, 62.0% (101) for US (*p* = 0.56), 45.2% (71) for FFDM (*p* < 0.0001) and 69.9% (114) for MRI (*p* = 0.28); underestimated in 17.4% for CESM, 19.6% for US (*p* = 0.56), 24.2% for FFDM (*p* = 0.03) and 6.7% for MRI (*p* = 0.0005); and overestimated in 16.2% for CESM, 16.6% for US (*p* = 1.00), 16.6% for FFDM (*p* = 1.00) and 22.7% for MRI (*p* = 0.02).

In the evaluation of the relationship between detection rate and size estimation efficacy of each technique, in respect to the traditional pathological characteristics, the detection rate was influenced by the confound variables only in the case of FFDM, where the prevalence of missed lesions significantly increased in smaller (pT1, 19.4%; pT2, 8.5%; pT3/4, 0%) and better differentiated (G1, 17.9%; G2, 20.0%; G3, 3.7%) tumors. With regard to size, underestimation with CESM was significantly more frequent in larger (pT1, 10.4%; pT2, 25.5%; pT3/4 40.0%; *p* = 0.001), multifocal (unifocal, 10.5%; multifocal, 34.0%; *p* < 0.01) and lobular (ductal, 12.3%; lobular, 35.0%; *p* = 0.02) tumors.

## Discussion

The rate of unnecessary intervention for breast lesions considered suspicious at radiological examination has increased over the last decade [18]. Furthermore, although the radiological methods currently used for early BC detection have contributed to a significant reduction of mortality, they are characterized by some degree of inconsistency. In particular, standard mammography is the only screening modality shown to reduce BC mortality rates, but its sensitivity and specificity can be affected by breast tissue density. The diagnostic efficacy of US, the imaging technique most commonly used in combination with FFDM, is closely related to operator experience. Finally, while MRI is the most sensitive breast imaging method, being capable of detecting tumor angiogenesis through contrast enhancement [19–23], it is affected by high rates of false positive results [20–23]. In this regard, CESM, based on the differential enhancement of neoplastic and normal tissue and coupling sensitivity to specificity, could represent a novel reliable tool for early detection of breast tumors [7–15].

It has been argued that the adoption into practice of CESM could be hampered by increased radiation exposure compared with FFDM and reactions to contrast medium. CESM is based on intravenous injection of an iodinated contrast reagent and mammography exposures in standard views at low (conventional) and high energies. Two images are used for reporting each view: the low energy and a combination of the low and high-energy images that corresponds to a “subtracted” iodine image. Low energy exposures are taken using standard FFDM techniques such that the resulting images and radiation dose are very similar to those obtained with FFDM. The high-energy image is adapted to impart a minimal dose; as a consequence, the total CESM examination dose is about 20% higher than a standard FFDM examination, but still within the EUREF Guidelines levels for digital mammography screening [16]. In keeping with previous data reporting a 0.2–0.4% risk, we did not observe any severe adverse reaction [24].

In this study, we evaluated the efficacy of CESM in a well characterized series of dense breast patients with histologically proven malignant mammary lesions, comparing its diagnostic performance with US, FFDM and MRI**.** We found that CESM properly detected 165 of the 176 lesions analyzed overall, yielding to a 93.7% detection rate, in turn similar to US and MRI and significantly higher than FFDM. CESM sensitivity was particularly high in invasive tumor patients, where it correctly detected all except three cases, corresponding to a 98% detection rate in this subgroup. A high sensitivity of CESM has previously been reported by other groups [7–10]. In particular, Dromain et al. [7] found CESM to have better sensitivity (93% *vs* 90%) and specificity (63% versus 47%) compared with FFDM plus US. The authors also showed that CESM correctly estimated tumor size, using the final histologic report as a reference.

Likewise, Jochelson et al. [10], who examined 52 women with newly diagnosed breast carcinomas, reported that CESM equaled MRI sensitivity, with a significantly lower false positive rate (2 vs 13 cases in CESM and MRI, respectively). These data were reproduced by Fallenberg et al. [8, 9], who also reported that CESM was more accurate than MRI in lesion size estimation; Fallenberg et al. also reported the correct estimation of lesion tumor size in two recent studies on 118 patients and 178 patients [9, 11].

CESM sensitivity was less than 50% for the 15 in situ lesions seen in our cohort. These were more efficiently detected by FFDM and MRI (approximately 80% detection rate each). This is in contrast to Lalji et al. [25], who have recently reported that the low-energy CESM images are non-inferior to FFDM images. Our results are not entirely surprising, taking into account that non-invasive lesions are usually associated with tissue microcalcifications, which are efficiently detected by FFDM.

Kuhl hypothesized the high sensitivity of MRI in identifying non-invasive lesions could be attributed to contrast media extravasation and accumulation around intraductal proliferations [26]. This process does not occur using the iodinated media in CESM analysis, as reported in previous studies focusing on CT scan methods [27–29].

Tumor size estimation plays a pivotal role in guiding surgical management in breast cancer patients [30–36]. Our data provides evidence that CESM combines high detection rates for invasive lesions with accurate size estimation. CESM assessment of invasive tumor size was concordant with pathological data in 64.6% of lesions, a figure similar to that obtained by US and MRI, but significantly higher than FFDM (*p* < 0.0001). The cases in which CESM underestimated cancer size (17% of the cases) consisted mainly of lobular BC, large tumors (pT3/4) and multifocal BC. MRI has previously been reported to be more efficient than CESM in detecting multifocal, multicentric, and synchronous tumors [10], but with a significantly increased false positive rate, consequently the CESM could not suffer from typical MRI clinical limitations.

Iodinated contrast media in highly vascularized tissues, provides an enhancement effect stronger than in normal surrounding breast tissue [20–23]. It is therefore tempting to speculate that CESM sensitivity largely relies on tumor vascularization [37]: interestingly, neo-angiogenesis has been reported to occur heterogeneously in lobular carcinomas in studies assessing micro-vessel density by immunohistochemistry for endothelial markers [38]. Collectively, these data suggest that CESM could actually be less sensitive in properly addressing tumor size in large, multifocal lobular carcinomas.

In our study, performed on large cohort of patients, we confirm that CESM provides additional information with consistent improvement of the cancer diagnosis in dense breasts and assessment in tumor size.

The major limitation of our study is that all patients in our cohort had dense breasts and presented with radiologically suspicious mammary lesions that were subsequently histologically diagnosed as malignant. These inclusion criteria may have led to a selection bias and consequently, that our results should be translated with caution to an unselected, radiologically “naïve” population. Moreover, this design meant that we were not able to retrieve data about CESM specificity.

Also, we were not able to assess the ability of CESM to evaluate in situ lesion dimensions, since available pathological data were discontinuous. Furthermore, the number of in situ lesions was too small to draw statistically significant conclusions about any putative difference in detection rate among different intraductal carcinoma subtypes.

Our data prompt us to hypothesize that CESM could enter into clinical practice for its sensitivity in early detection of breast tumors, as a valid adjunct in the surgical management of BC patients [39]. Further studies are needed to define the future role of CESM in breast cancer staging and management.

## Abbreviations

CESM: Contrast enhanced spectral mammography
BC: Breast cancer
US: Ultrasound
MRI: Magnetic resonance imaging
FFDM: Full field digital mammography
BI-RADS: Breast imaging reporting and data system
VABB: Vacuum-assisted breast biopsy
CC: Cranio-caudal
MLO: Medio-lateral-oblique
